# Stability of A Coevolving Host-parasite System Peaks at Intermediate Productivity

**DOI:** 10.1371/journal.pone.0168560

**Published:** 2017-01-11

**Authors:** Xin-Feng Zhao, Yi-Qi Hao, Quan-Guo Zhang

**Affiliations:** State Key Laboratory of Earth Surface Processes and Resource Ecology and MOE Key Laboratory for Biodiversity Science and Ecological Engineering, Beijing Normal University, Beijing, China; Charles University, CZECH REPUBLIC

## Abstract

Habitat productivity may affect the stability of consumer-resource systems, through both ecological and evolutionary mechanisms. We hypothesize that coevolving consumer-resource systems show more stable dynamics at intermediate resource availability, while very low-level resource supply cannot support sufficiently large populations of resource and consumer species to avoid stochastic extinction, and extremely resource-rich environments may promote escalatory arms-race-like coevolution that can cause strong fluctuations in species abundance and even extinction of one or both trophic levels. We tested these ideas by carrying out an experimental evolution study with a model bacterium-phage system (*Pseudomonas fluorescens* SBW25 and its phage SBW25Φ2). Consistent with our hypothesis, this system was most stable at intermediate resource supply (fewer extinction events and smaller magnitude of population fluctuation). In our experiment, the rate of coevolution between bacterial resistance and phage infectivity was correlated with the magnitude of population fluctuation, which may explain the different in stability between levels of resource supply. Crucially, our results are consistent with a suggestion that, among the two major modes of antagonistic coevolution, arms race is more likely than fluctuation selection dynamics to cause extinction events in consumer-resource systems. This study suggests an important role of environment-dependent coevolutionary dynamics for the stability of consumer-resource species systems, therefore highlights the importance to consider contemporaneous evolutionary dynamics when studying the stability of ecosystems, particularly those under environmental changes.

## Introduction

The stability of consumer-resource species systems (including predator-prey, parasite-host and herbivore-plant systems) has attracted continuous research interest in ecology and evolution [1–9]. The classical oscillation dynamics predicted by ecological models may be altered by numerous factors, with stability changed in both quantitative (strength of population fluctuation) and qualitative senses (persistence versus extinction of one or both trophic levels). Those factors include reduction of host reproductive potential induced by parasite, time delays of reproduction and transmission of parasite [10], host stage structure [7] and habitat productivity [5]. Contemporaneous coevolution was added to this list in recent years; for example, evolution of prey defense against predators was found to produce long-period fluctuation in predator and prey densities and thus promote stability [11, 12]. This could be a scenario where coevolution leads to the coexistence of consumer and resource species that consist of both susceptible and resistant genotypes [4, 13–15]. However, both theoretical and experimental research also demonstrated alternative scenarios including persistent population cycles or extinction of consumers [1, 8, 16]. The effects of coevolution on resource-consumer system stability may largely depend on the mode and strength of coevolution that are shaped by a variety of environmental factors [6, 8, 17–19].

Here we address the importance of habitat productivity, which has long been considered as an important ecological driver of consumer-resource system stability [5, 20]. Its impact on coevolutionary dynamics and population stability has also received attention [8, 21–23]. For example, a recent experimental evolution study with a bacterium-phage system found that resource enrichment may increase the chance of consumer species extinction by leading to highly asymmetrical coevolution (that is, extremely high-level of defense of resource species against consumers) [23]. In the present study we suggest that considering a broader range of habitat productivity lead to a prediction that intermediate productivity allows for the most stable dynamics in coevolving resource-consumer systems. Extremely low-resource environments cannot maintain sufficiently large populations [20], particularly for the consumer species when evolution of defense of resource species further decrease the growth performance of consumers. On the other hand, very high-level resource availability may also destabilize resource-consumer systems through evolutionary mechanisms. High resource supply can accelerate coevolution because of its effects in increasing population sizes (and thus the supply of mutations) and decreasing fitness costs associated with defense traits [21, 22]. Previous studies showed that greater resource availability may support continuous arms race dynamics (ARD) coevolution, while in low-resource environments fluctuating selection dynamics (FSD) are more common [21]. The two different modes of coevolutionary dynamics, FSD and ARD may show substantially different impacts on population stability. FSD occur when genotype frequencies in consumer and resource species undergo oscillations driven by frequency-dependent selection, allowing for the maintenance of within-population genetic diversity [24–27]. Under FSD, populations may show stable demography, with cycled selection on a limited number of alternative genotypes [11, 13, 25, 28]. By contrast, ARD arise where genotypes in consumer and resource species undergo successive selective sweeps driven by directional selection for increased defense and counter defense over time; and the maintenance of ARD requires a continuous supply of new mutations [29, 30]. Therefore an association between ARD and higher probability of consumer extinction is expected.

We tested these ideas using laboratory populations of the bacterium *Pseudomonas fluorescens* SBW25 [31] and its phage SBW25Φ2 [32]. Previous studies of this system documented almost perfect escalation of bacterial resistance and phage infectivity over time during short-term interactions [32, 33], and habitat productivity had an influence on the coevolutionary dynamics [21, 22]. While those previous studies reported phage extinction events only very occasionally [34], we often observed phage extinction in our laboratory, which was driven by bacterial resistance evolution. Accordingly, we set up microcosms where bacteria and phage coevolved under three resource levels. We predicted that the coevolving population at intermediate resource supply should have both lower levels of population fluctuation and smaller chance of population extinction, while in high- and low-resource environments this systems may show less stable dynamics due to intense coevolutionary dynamics and small carrying capacity, respectively.

## Materials and Methods

### Selection experiment

The bacterium *Pseudomonas fluorescens* SBW25 [31] and its lytic bacteriophage virus SBW25Φ2 [32] were used in this study; both of which were provided by Angus Buckling. Bacteria and phages were grown as batch cultures in static microcosms of 1.5 mL of nutrient media in 24-well microplates at 28°C. We used three culture media, referred to as KB, 0.1KB and 0.01KB. The three media were made by supplementing M9 buffer (6 g L^-1^ of Na_2_HPO_4_, 3 g L^-1^ of KH_2_PO_4_, 1 g L^-1^ of NH_4_Cl, 0.5 g L^-1^ of NaCl) with different amounts of nutrients (10 g L^-1^ of glycerol and 20 g L^-1^ of proteose peptone no. 3 for KB, while 0.1- and 0.01-fold of the nutrients for 0.1KB and 0.01KB, respectively). Higher resource supply could significantly increase bacterial growth performance (S1 Fig).

Twelve replicate microcosms were set up under each level of resource supply. Each microcosm was inoculated with ~10^6^ bacterial cells that had been starved in M9 buffer for 5 h following overnight growth in KB broth, and ~10^4^ phage particles. Cultures were then propagated for 20 serial transfers (one transfer every two days). At every transfer, each culture was transferred to a centrifuge tube and vortexed, 15 μL (1%) of which was then transferred to a fresh microcosm. Isolation of phages from cultures was achieved by mixing 450 μL of culture with 50 μL of chloroform, which was then vortexed to lyse the bacterial cells, and centrifuged at 15,800g for 2 min to pellet the bacteria debris, leaving a suspension of phage in the supernatant. Phage density was measured at each transfer by plating phage dilutions onto soft agar plates containing the ancestral bacterial cells and counting the number of plaque forming units (PFUs) after 24 h incubation. Bacterial density of every evolution line was measured as optical density by an automated microplate reader (Bio-tek Elx800-NB) until phage extinction.

### Inference of coevolutionary dynamics

Coevolutionary dynamics were inferred by measures of bacterial resistance to phages. We measured the resistance of bacterial populations from four transfers in the past, contemporary transfer, and four transfers in the future to the phage population at a given point in time (e.g., bacteria from transfer 4, 8 and 12 were challenged by phages from transfer 8). The difference in resistance provides a measure of the evolution of bacterial resistance over this period of eight transfers. In the same way, the evolution of phage infectivity was measured as change of resistance of bacterial population at the contemporary transfer to phages through time [22, 33]. To assay the resistance of a bacterial population against a given phage population, we randomly isolated 20 bacterial colonies, suspensions of which were streaked across a line of phage (20 μL) that had previously been streaked and dried on KB agar plates. A colony was defined as sensitive if its growth was inhibited, otherwise it was scored as resistant. An ancestral bacterial colony was used as a control for each assay (a failure of infection of the ancestral bacterium by a phage population indicated very low phage density and thus unreliable measurement of bacterial resistance).

### Statistical analysis

Duration of persistence (time to extinction) of phage lines was analyzed using the parametric survival regression model (with the default Weibull distribution), where resource availability was included as a fixed factor. In addition, unconditional exact test [35] was performed for the independence between phage extinction and resource availability. We also compared the stability of systems between environments during the first eight transfers (early stage of coevolution) and the later twelve transfers (late stage of coevolution) separately by using unconditional exact test.

Fluctuation of populations was also estimated, calculated as population density change for each phage and bacteria line at each transfer, as | D_t_−D_t-1_ |, where D_t_ = log_10_ (PFU mL^-1^ +1) for phage and D_t =_ log_10_ (OD_600nm_) for bacteria, representing the population density at transfer t. The population density change values were analyzed with mixed-effect linear model. Resource supply level was fitted as a fixed effect, time (transfer number) as covariate, and evolution line ID as a random factors. Microcosms of 0.01KB were excluded from this analysis due to very early phage extinction in this environment.

We investigated the possibility that coevolutionary dynamics can explain the effect of productivity in population stability. The evolution of phage infectivity and bacterial resistance in KB and 0.1KB environments were analyzed with mixed-effect linear model, where resistance data were arcsine-transformed as response variable, resource level was included as a fixed explanatory variable, the contemporary transfer and relative time (four transfers in the past, contemporary, four transfers in the future) as covariates, evolution line ID as a random factor. All statistical tests were carried out in the R environment [36].

## Results

### Greater chance of phage persistence in intermediate-resource microcosms

**Fig 1:**
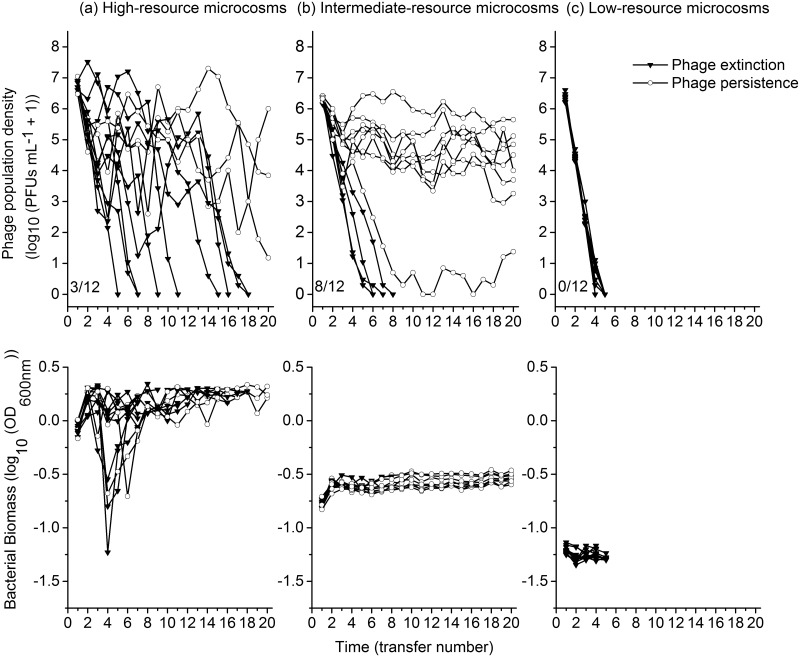
Phage and bacterial densities in microcosms through time. Phage and bacterial densities over time in microcosms of high- (column a), intermediate- (column b) and low-resource microcosms (column c). Each line indicates an individual phage or bacterial line. The numbers in panels of the first row indicate the frequencies of phage populations that persisted until the end of the experiment.

Among the 36 evolution lines, eleven observed phage persistence until the end of experiment. Phage populations went extinct in all the twelve low-resource microcosms, but persisted in eight out of twelve intermediate-resource microcosms, and three out of twelve high-resource microcosms (Fig 1). The intermediate-resource supply showed lower probability of phage extinction than the high-resource environment (4/12 marginally non-significantly lower than 9/12 for the whole 20 transfers, unconditional exact test, P = 0.064) and low-resource supply (4/12 significantly lower than 12/12, unconditional exact test, P < 0.001). Consistent results were obtained from survival analysis (Fig 1; survival regression model, intermediate-resource supply compared to high-resource supply, *z* = -1.83, *P* = 0.067, intermediate-resource supply compared to low-resource supply, *z* = -7.95, *P*< 0.001; where the scale parameter of survival regression model 0.363, less than 1, indicated that the extinction rate of phage decreased with time).

### Both phage and bacterial populations showed weaker fluctuation in intermediate-resource microcosms

**Fig 2:**
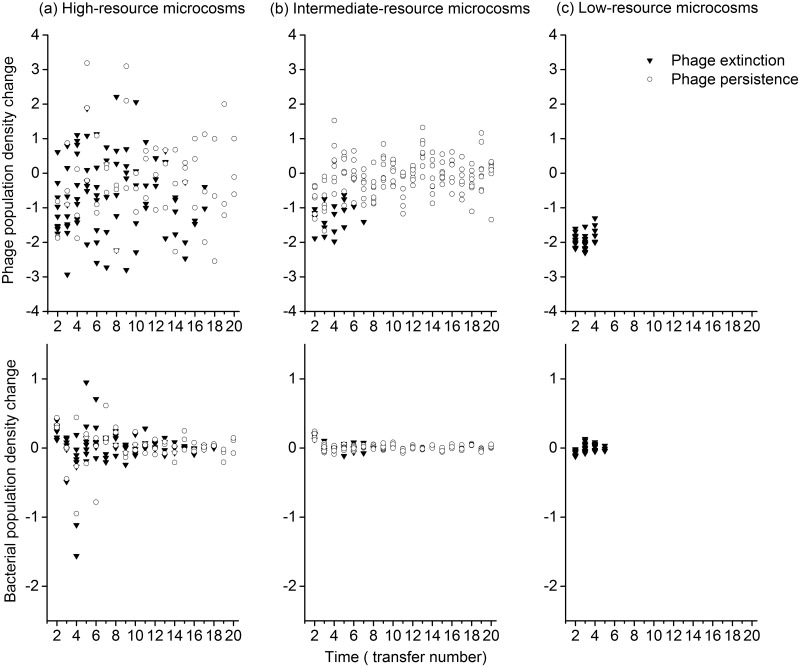
Change in phage and bacterial population densities. Change in phage (top panels) and bacterial (bottom panels) population densities (D_t_−D_t-1_, where D_t_ = log_10_ (PFU mL^-1^ +1) for phage and D_t =_ log_10_ (OD_600nm_) for bacteria, representing the population density at transfer t) of each line through time in high- (column a), intermediate- (column b) and low-resource microcosms (column c). The positive values indicate the increasing of population density. The negative values indicate the decreasing of population density.

Phage populations in low-resource microcosms declined consistently to extinction, while fluctuation over time was observed in high- and intermediate-resource microcosms (Fig 2). The extent of phage population change per transfer was smaller in intermediate- than high-resource environment (mixed-effect linear model, resource levels, F_1,22_ = 33.721, P < 0.001), and decreased over time in both environments (time, F_1,290_ = 12.974, P < 0.001).

Bacteria persisted in all microcosms. The extent of bacterial population change between consecutive transfers was larger in high- than intermediate-resource environment (Fig 2; mixed-effect linear model, resource levels, F_1,22_ = 15.305, P < 0.001); and decreased over time in both environments (time, F_1,303_ = 44.772, P < 0.001), with a large-magnitude decline in high-resource microcosms (resource levels × time, F_1,303_ = 28.629, P < 0.001).

### Coevolutionary rate was greater in high-resource microcosms

**Fig 3:**
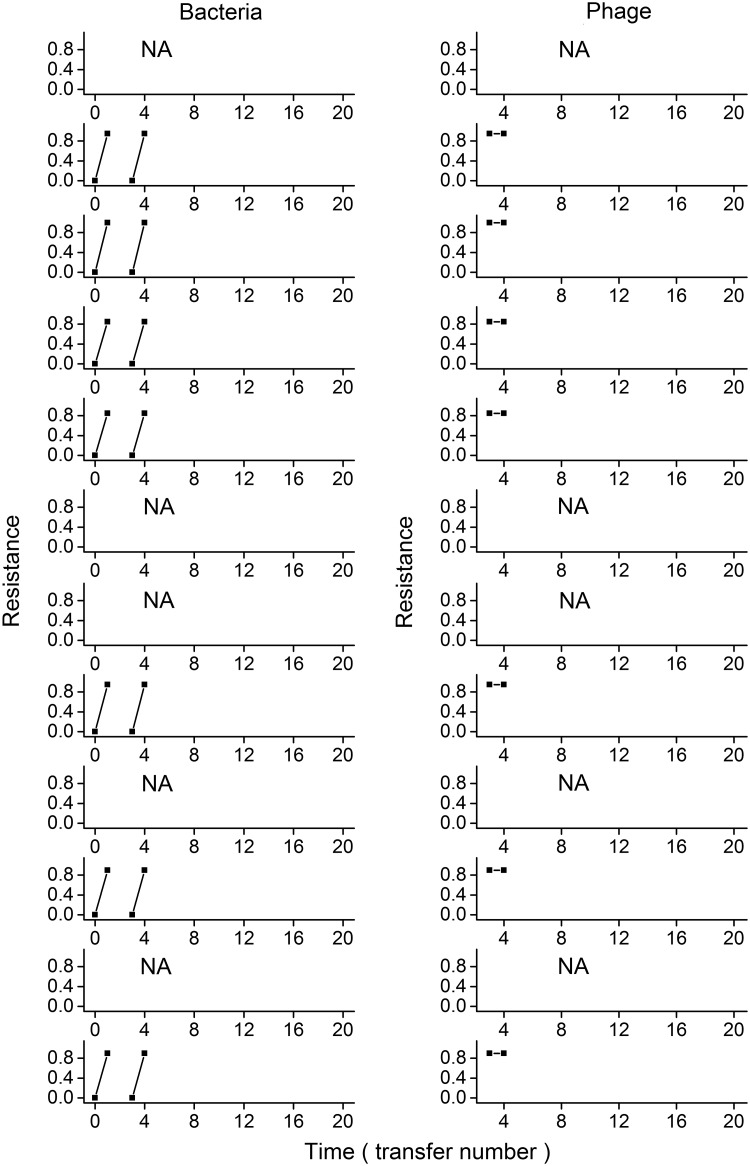
Change in bacterial resistance and phage infection in low-resource microcosms. Each row represents one individual microcosm. In each panel of the left column, each line shows the proportion of bacteria from four transfers in the past, contemporary transfer and four transfers in the future (from left to right) that are resistant to phages from a given transfer. A positive slope indicates an increase in bacterial resistance over time. In each panel of the right column, each line shows the proportion of bacteria from a given transfer that are resistant to phages from four transfers in the past, contemporary transfer and four transfers in the future (from left to right). A negative slope indicates an increase in phage infection over time.

**Fig 4:**
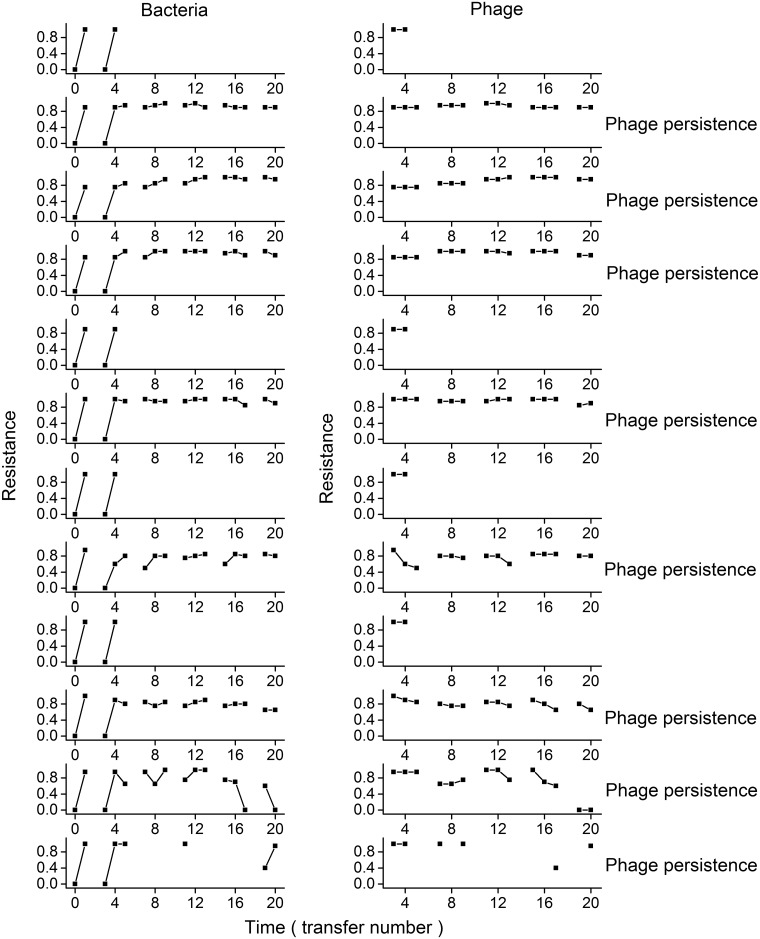
Change in bacterial resistance and phage infection in intermediate-resource microcosms. Symbols are as in Fig 3.

**Fig 5:**
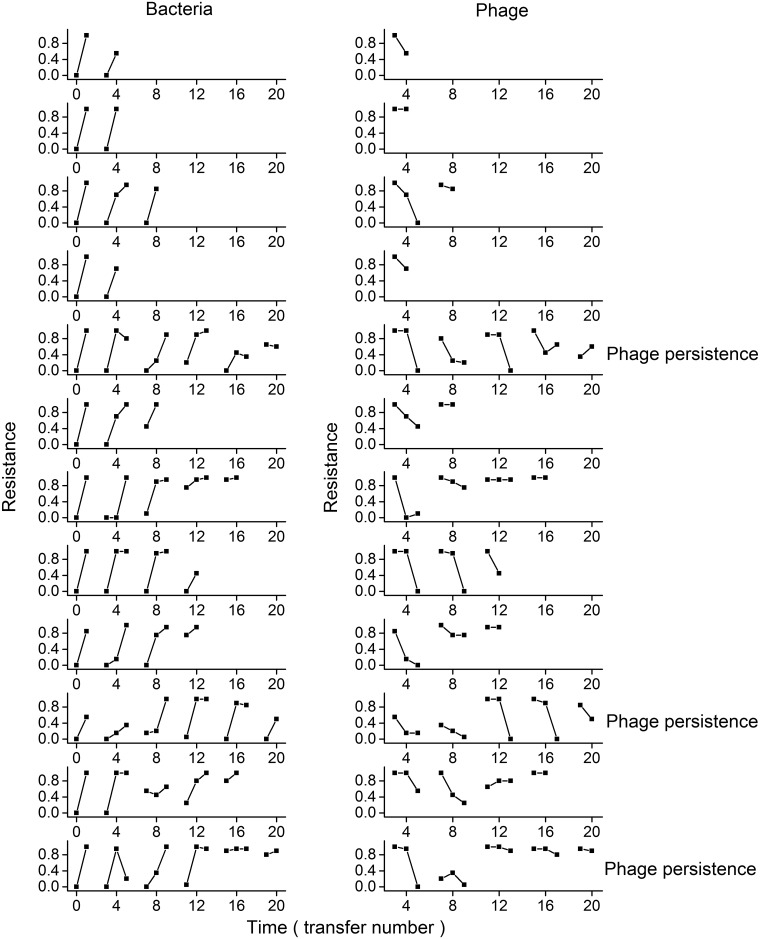
Change in bacterial resistance and phage infection in high-resource microcosms. Symbols are as in Fig 3.

Short-term increases in the phage infectivity and bacterial resistance indicated the evolution of infectivity and resistance. Low-resource microcosms showed increases in bacterial resistance over time and unchanged phage infectivity (Fig 3). In high- and intermediate-resource microcosms, phage and bacteria showed coevolution (Figs 4 and 5; mixed-effect linear model, evolution of bacterial resistance, relative time, F_1,224_ = 122.398, P < 0.001; evolution of phage infection, relative time, F_1,175_ = 60.092, P < 0.001); the coevolution rate was faster in high- than intermediate-resource environment (evolution of bacteria, resource levels× relative time, F_1,224_ = 20.664, P < 0.001; evolution of phage, resource levels× relative time, F_1,175_ = 51.217, P < 0.001), and decreased over time in both environments (evolution of bacteria, transfer× relative time, F_1,224_ = 45.917, P < 0.001; evolution of phage, transfer× relative time, F_1,175_ = 8.7571, P = 0.004).

## Discussion

Knowledge of the environmental determinants of coevolutionary dynamics in consumer-resource species interactions and the consequences for food web stability is crucial for our understanding of the maintenance of biodiversity, particularly for microbes that have the potential of rapid evolution under environmental changes [8, 11, 12, 23, 37, 38]. Here we work with a bacterium-phage system to test a hypothesis that coevolving host-parasite systems were most stable in intermediate-resource environment.

Our choice of the experimental system, bacterium *P*. *fluorescens* SBW25 and its lytic phage SBW25Φ2, was based on (i) an earlier finding that resource supply can affect coevolutionary dynamics of this system [21, 22], and (ii) the observation that the phage parasite more often go extinct during coevolution in our laboratory (at Beijing) than found by previous studies most of which were conducted at Oxford [22, 32, 33, 39, 40]. The reason for this difference between laboratories is unclear; and two possibilities are as the follows. First, the phage strain used in the present study may have accumulated certain mutations that limited its evolution in infectivity (mutation accumulation could have occurred as we renewed our phage stocks every two years, when new cultures were prepared from frozen samples and stored, replacing the old ones). Second, some uncontrollable differences in lab environmental factors such as water quality may affect ecological and evolutionary dynamics [41].

In our experimental system, phage extinction have been driven by bacterial resistance evolution that decreases phage growth. The phage may evolve broader infectivity range as a response to bacterial resistance evolution. However, it might be easier for bacteria to evolve resistance against phages than for phages to overcome this resistance, as mutations in the bacteria that alter receptors on the bacterial cell surface to confer resistance might be more accessible than mutations in the phages that allow new ‘match’ to the alteration to become infective [42]. Earlier observations also showed the phage populations often had more limited potential of evolution relative to the bacteria [39, 40]. Note that serial transfers caused 100-fold dilution disturbances and could increase the extinction risk of phage lines. However, we suggest this should not be the ultimate driver of phage extinction, because of (i) an earlier finding that phage populations could be maintained at constant sizes through time when continuously evolving with susceptible bacteria [43], and (ii) an observation in the present study that every phage line showed a population increase of > 100-fold at the beginning of the experiment (from ~10^4^ at inoculation to > 10^6^ mL^-1^ at transfer 1) when the bacterial populations were dominated by susceptible genotypes.

We found distinct phage extinction patterns under three resource environments: (i) all phage lines in low-resource environment (0.01KB) went extinct rapidly; (ii) there were more persistent lines under intermediate- than high-resource supply; (iii) phage extinction events in intermediate-resource environment only occurred during the early stage of coevolution, while phage extinction events in high-resource microcosms occurred during both early and late stages. Dependence of coevolutionary dynamics on resource supply could well explain these findings. Phage persistence in this coevolving system is most likely if (i) the phage could achieve some extent of infectivity evolution to overcome the initial step(s) of bacterial resistance evolution, and (ii) further escalation of bacterial resistance is limited by fitness costs associated with generalism in resistance [26, 27]. Those early extinction events, especially in the low- and intermediate-resource environments, were due to a lack of phage infectivity evolution to overcome the evolution of bacteria resistance under arms-race-type coevolution during early stage of experiment. Fitness costs of resistance may prevent bacteria to evolve very high levels of resistance in intermediate-resource environment during the late stage of the experiment. In a number of intermediate-resource microcosms the evolution of phage and bacteria slowed down, showed little changes in infectivity and resistance after transfers 8. Therefore, the intermediate-resource microcosms may reach the equilibrium of coevolution or FSD, which could stabilize the consumer-resource systems [26, 44], leading to the significantly less phage extinction events in intermediate- than high-resource environment during the late stage of coevolution (0/8 significantly lower than 6/9 during the last twelve transfers, unconditional exact test, P = 0.004) and deceasing extinction rate through time in intermediate-resource microcosms. In the high-resource environment, however, fitness costs of resistance may be mitigated to some extent and rapid evolution of bacterial resistance can be maintained for longer periods of time with higher coevolution rate, which fully exposed the evolutionary disadvantage of phage against bacteria in this system, increasing the chance of phage extinction [1, 8, 23, 26]. Compared to DNA-based phage used in our experiment, RNA-based phages (e.g. phage Φ6 that infects other *Pseudomonas* species) with low-fidelity replication have higher mutation rate [45–47]. This character of RNA-based phages may benefit phage from avoiding the early extinction due to the limitation of infective mutations; however the high mutation rate can decrease the robust of evolvability with accumulation of deleterious mutation [48], which may cause the phage extinction in later stage, especially under strong antagonistic coevolution likely occurring in high-resource supply. Thus the same extinction patterns of phage along productivities may happened to RNA-based phages.

Besides phage extinction, fluctuations of both phage and bacteria populations were also weaker in intermediate- than high-resource environment. The fluctuation of abundance could increase the chance of stochastic extinction, especially when periodical disturbances such as serial 100-fold-dilution transfers occur [49–51]. Phage populations could be maintained at constant sizes through time when continuously evolving with susceptible bacteria [43], therefore fluctuation of phage populations was, at least partially, caused by the evolution of bacterial resistance. That was further confirmed by (i) an association between more rapid coevolution and stronger population fluctuation at early stage of experiment in the both high- and intermediate environments, and (ii) the larger fluctuations of phage and bacteria population in high-resource microcosms which were under higher rate of coevolution.

Our findings indicate the different stability of host-parasite system under coevolution across three levels of habitat productivity, and highlight that the effects of habitat productivity on the mode of resource-consumer coevolution have importance consequences for food web stability. It would be of great interest for future studies to investigate the link between rapid evolutionary processes with ecological processes that determine the stability of consumer-resource systems, and the effect of contemporaneous coevolution on population stability in heterogeneous environments.

## Supporting Information

S1 Fig Population sizes of bacteria grown in three different levels of resource supply. (DOCX)
