# Supplementary material for: Stability of A Coevolving Host-parasite System Peaks at Intermediate Productivity
Source: PLoS One. 2017 Jan 11;12(1):e0168560. doi: 10.1371/journal.pone.0168560 (PMC5226335; doi:10.1371/journal.pone.0168560)

**Stability of a coevolving host-parasite system peaks at intermediate productivity**

**Supporting information**

**S1 Fig.** **Biomass of bacteria without phage in three different levels of resource supply.**

Bacterial population size, measured as optical density (at 600nm), after 48 h of growth without phage in three different levels of resource supply. Data show mean ± SE (n = 12). Bars with different letters differ significantly from each other (Tukey contrasts, *P*_adj_< 0.001).


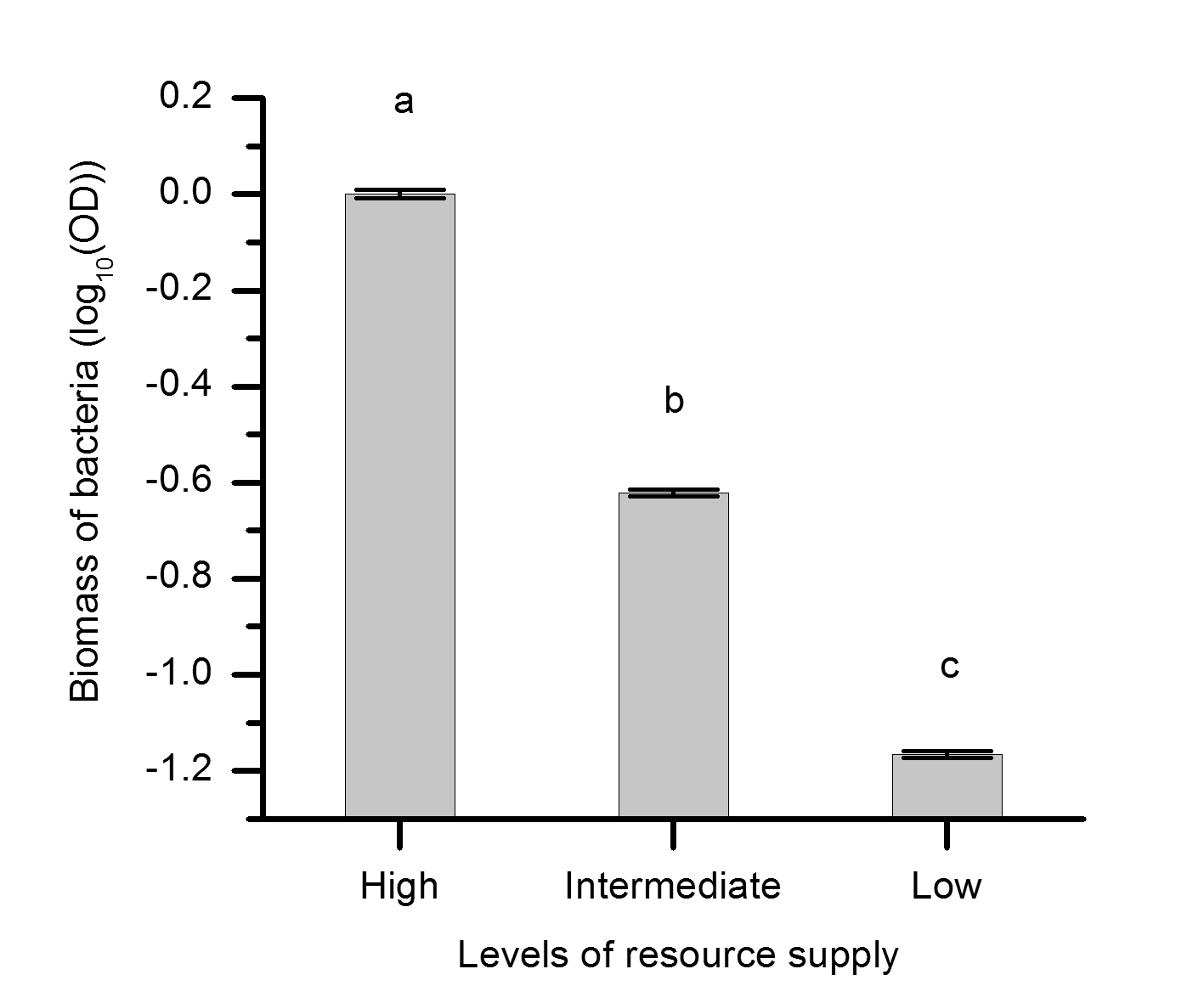

Supplement: S1 Fig — (DOCX) [file pone.0168560.s001.docx]
